# Strangulated Inguinal Hernia Containing Caecum and Inflamed Appendix: Two Case Reports and Review of the Literature to Guide Management

**DOI:** 10.3389/jaws.2025.15273

**Published:** 2026-01-09

**Authors:** Guillaume Tcheutchoua Soh, Papa Mamadou Faye, Armel Franck Tene Nde, Abdourahmane Ndong, Thierno Amadou Telly Diallo, Nedra Jaouahdou, Abdoul Kharim Diop, Jacques Noel Tendeng, Ousmane Thiam, Philippe Manyacka Ma Nyemb, Alpha Oumar Toure, Ibrahima Konate, Mamadou Cisse

**Affiliations:** 1 Department of Surgery, Gaston Berger University, Saint-Louis, Senegal; 2 Department of Surgery, Universite Cheikh Anta Diop Bibliotheque Universitaire, Dakar, Senegal; 3 Department of Surgery, Universite Cheikh Anta Diop de Dakar, Dakar, Senegal

**Keywords:** Amyand hernias, appendicitis, desarda, losanoff-basson, strangulated hernia

## Abstract

**Introduction:**

Amyand’s hernia is defined by the presence of the appendix in the sac of an inguinal hernia. Its treatment poses two problems: the risk of infection associated with the presence of the appendix and the choice of surgical technique, which must reduce the risk of infection while minimising the risk of recurrence. In this article, we present two patients treated for a strangulated inguinal hernia containing the appendix and caecum and review the literature for evidence that may guide the choice of treatment.

**Case Presentation:**

We present two male patients, aged 37 and 44, admitted to our department for a strangulated right inguinal hernia. During surgical exploration, the hernia sacs of both patients contained the appendix and caecum, which were inflamed without necrosis. Both patients underwent appendectomy with protection of the surgical site and instillation of povidone-iodine into the surgical site to reduce the risk of infection. Both were treated using the Lichtenstein procedure. The postoperative course was uneventful in one patient, while the other had oedema of the cord, which responded well to enzymatic anti-inflammatory treatment made of on trypsin, pancreatic ribonuclease and chymotrypsinogen. Examination of the surgical specimens confirmed appendicitis.

**Conclusion:**

Most Amyand hernias are diagnosed during the treatment of a complicated hernia. There are no specific signs, hence the importance of systematically opening the hernia sac when treating a complicated hernia. During surgery, the surgeon must choose an appropriate technique based on the risk of infection and perform a routine appendectomy, followed by an examination of the surgical specimen. The role of hernioplasty according to the Desarda procedure could be evaluated for this indication. The Losanoff-Basson classification should be constantly updated as treatment procedures and available resources evolve.

## Introduction

Amyand’s hernia is defined by the presence of the appendix in the inguinal hernia sac. It is a rare type of inguinal hernia with an unknown prevalence in the general population, which can be discovered during scheduled hernia repair or emergency hernia surgery [1]. Based on the evolution in the surgical treatment of inguinal hernia, its current treatment according to Losanoff-Basson classification poses two fundamental problems:

First, the presence of the appendix in the hernia sac may appear normal or pathological [1, 2]. Appendicular pathology has the particularity of having several degrees of infectious risk depending on the stage of the pathology. Thus, it may be a type 2 (Clean, Contaminated) surgery according to Altemeir’s classification of infectious risks in the case of a normal or catarrhal appendix, or be classified as type 4 (Dirty or Infected) in the case of perforation with the presence of pus in the hernia sac [1, 3]. This concept of infection in Amyand’s hernia has been taken into account in the Losanoff-Basson classification (Table 1), which facilitates understanding of the risk of infection and other possible complications in cases of Amyand’s hernia [1, 4]. However there is no consensus for appendectomy even for type 1 Losanoff-Basson Amyand’s hernia [5].

**TABLE 1:** Pathological types of Amyand’s hernia and their respective management, Losanoff-Basson 2008 [4].

Type of hernia	1	2	3	4
Salient features	Normal appendix	Acute appendicitis localized in the sac	Acute appendicitis, peritonitis	Acute appendicitis, other abdominal Pathology
Surgical management	Reduction or appendectomy (depending on age), mesh hernioplasty	Appendectomy through hernia, endogenous repair	Appendectomy through laparotomy, Endogenous repair	Appendectomy, diagnostic workup and other procedures as appropriate

Secondly, modern hernia treatment is based on the absence of tissue tension during hernia repair. Over time, tension-free techniques have shown a drastic reduction in recurrence rates and improved patients' quality of life [6]. Most tension-free techniques use a mesh that can be placed using several methods, which aim to reinforce the posterior wall of the inguinal canal. The current gold standard for these techniques is the procedure described by Irving Lichtenstein in 1984 [7]. The main risk associated with the use of prostheses is infection, which may require excision of the prosthesis in extreme cases. Other techniques, such as the Desarda procedure, allow for tension-free repair without the need for a prosthesis [8]. In sub-Saharan Africa, the Bassini procedure, which is a tension repair technique criticised for its high recurrence rate, is still the most widely used. It has the advantage of a low risk of infection, is simple to perform and easy to learn [9]. We hereby question the place of pure tissue tension-free procedures in the management of Amyand’s hernia.

In this article, we present two patients treated for strangulated inguinal hernias containing the appendix and caecum and review the literature for evidence that may guide the choice of surgical treatment. The study was reported according to the CARE guidelines [10].

## Case Description

### Case 1

**FIGURE 1:**
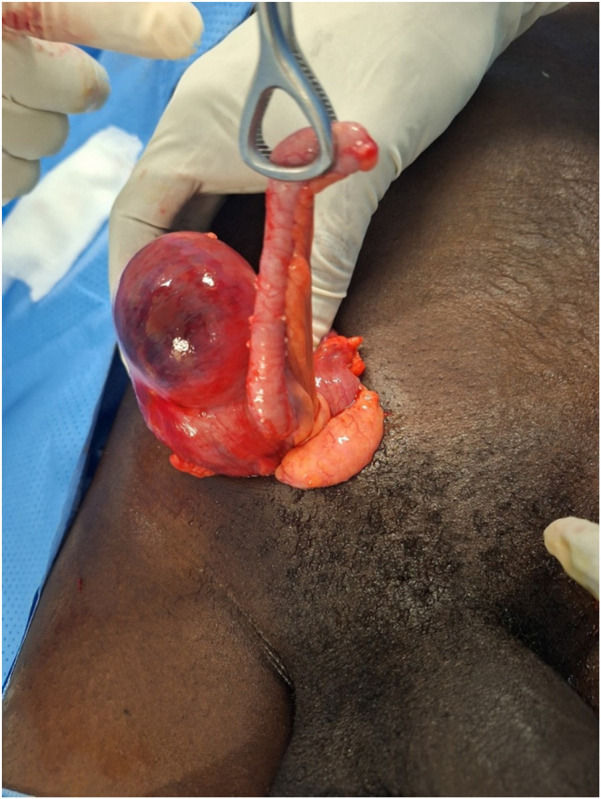
Appendix and caecum into the hernia sac after opening, case 1.

This is a 37-year-old patient who works as a farmer and has had a swelling in his right groin for 1 year. He was admitted to our department with pain in his right groin, constipation and flatulence for 6 days, and vomiting for 2 days. His vital signs were normal on admission. On examination, he had abdominal distension, diffuse tympanism, a painful, irreducible swelling in the inguinal region and right scrotum, and the rectal ampulla was empty on palpation. In addition, there was an uncomplicated left inguinal hernia. We diagnosed a strangulated right inguinal-scrotal hernia. An abdominal X-ray was performed, which showed fluid levels. Laboratory tests revealed abnormalities including hyponatraemia at 122 mmol/L, hypochloraemia at 80.2 mmol/L and increased C-reactive protein at 24 mg/L. Blood counts were normal. The patient underwent preoperative resuscitation. Exploration was performed via a transverse right inguinal incision under general anaesthesia, and revealed an inflamed appendix and a viable ischaemic caecum. Figure 1. The patient was classified as type 2 according to the Losanoff-Basson classification. We protected the surgical field with povidone-iodine-coated compresses and performed an antegrade appendectomy. We pushed back the caecum, dissected the hernia sac from the spermatic cord elements and then resected the hernia sac. We inserted a polypropylene mesh using the Lichtenstein technique. The prosthesis was irrigated with povidone-iodine before closure to reduce risk of infection. The postoperative period was marked by persistent abdominal distension 24 h after surgery, which improved on day 2 after surgery. The patient received antibiotics based on amoxicillin and clavulanic acid (80 mg/kg/day), paracetamol (15 mg/kg/6 h) and nefopam (20 mg/8 h) in accordance with our local prescription protocol. The patient was discharged on the third day after surgery. The patient was reviewed on day 7. Examination revealed oedema of the spermatic cord. He was treated with an enzymatic anti-inflammatory drug (Ribatran^R^). On day 30 after surgery, the examination was normal. Pathological examination of the surgical specimen confirmed acute appendicitis. He underwent Lichtenstein repair on the left side 2 months after surgery with an uncomplicated postoperative recovery. The patient will be monitored during consultations if required.

### Case 2

**FIGURE 2:**
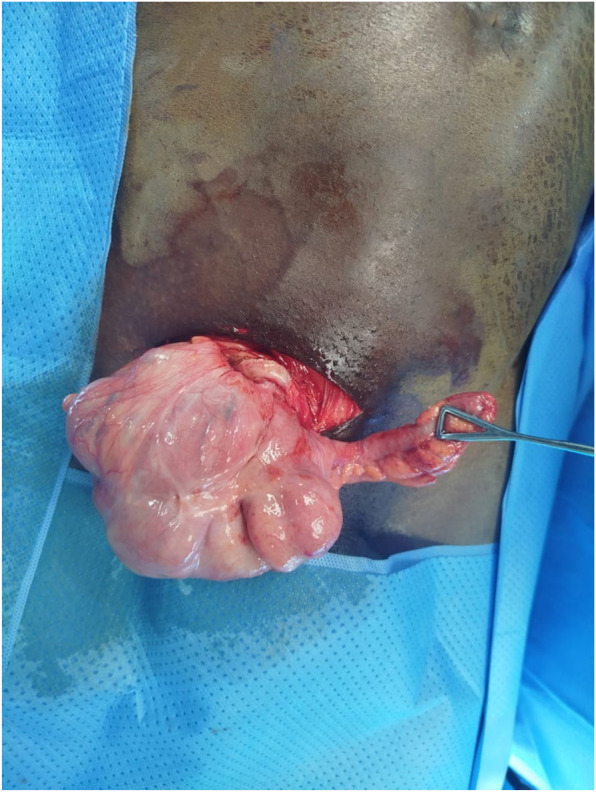
Appendix and caecum into the hernia sac after opening, case 2.

This is a 44-year-old male patient, a truck driver, who had had a right inguinal hernia for 2 years. He was admitted 3 days following the first patient. He came to the clinic for a right inguinal-scrotal swelling that had become painful and irreducible 11 h before the consultation, without vomiting or associated transit disorders. On physical examination, the abdomen was soft. The rectal ampulla contained faecal matter on examination. A strangulated right inguinal-scrotal hernia was diagnosed. Laboratory tests showed white blood cells at 5,200 and neutrophils at 78.6%. Urea, creatinine and electrolytes were normal. He underwent surgery via a transverse right inguinal incision under general anaesthesia. After opening the hernia sac, we discovered an inflamed appendix and a viable caecum. Figure 2. The patient was classified as type 2 according to the Losanoff-Basson classification. We protected the surgical site with povidone-iodine-coated compresses and performed an antegrade appendectomy. We pushed back the caecum before closing the hernia sac. A Lichtenstein repair was performed with a polypropylene prosthesis. The prosthesis site was irrigated with povidone-iodine before closure before closure to reduce risk of infection. The postoperative course was uneventful. The patient was discharged 24 h after surgery. The patient received antibiotics based on amoxicillin and clavulanic acid (80 mg/kg/day), paracetamol (15 mg/kg/6 h) and nefopam (20 mg/8 h) in accordance with our local prescription protocol. Follow-up on day 7 and day 30 after surgery was normal. Pathological examination of the surgical specimen confirmed acute appendicitis. The patient will be monitored during consultations if required.

## Discussion

Amyand’s hernia is a type of hernia characterised by the presence of the appendix in the hernia sac. It was first described by Claudius Amyand in 1736, at a time when most hernia treatments were based on tissue repair. The two cases observed in our study were young adults aged 37 and 44 years old. This age group was equally reported in Matanakis' systematic review, although most cases observed are in children and the older adults [1, 11].

The two cases we report were diagnosed at surgery for a strangulated hernia. Most Amyand hernias are diagnosed in emergencies [1, 12]. This may lead to an underestimation of the prevalence of this type of hernia, as opening the hernia sac is only systematic in complicated hernias. In contrast, the appendix may also be present in uncomplicated hernias [1]. The mechanism of occurrence of this hernia is not exactly known. However, It has been reported that, the patency of the processus vaginalis predisposed children to Amyand’s hernia more than adults [13]. We did not identify any causes in our two patients. Furthermore, this hernia can also occur on the left side (9.5%), resulting from an anomaly such as situs inversus, intestinal malrotation or a mobile caecum [1].

The diagnosis in our two patients was made during surgical exploration. Matanakis also report this in his systematic review, where the majority of cases (281/442) were operated on as emergencies [1]. Amyand’s hernia does not present any specific clinical signs. During emergencies, the clinical picture seems to oscillate between that of a complicated hernia and acute appendicitis. Furthermore, the presence of a tubular structure in the hernia sac attached to the caecum with hyperaemia is pathognomonic of an Amyand hernia, and the presence of the caecum near an hernia orifice is suggestive [1]. These data can be obtained by ultrasound and CT scan [14]. Routinely, we only perform imaging in cases of serious doubt about a strangulated hernia.

The Losanoff-Basson classification is a guide to the appropriate surgical procedure, as it is based on the severity of appendicitis and possible associated abdominal pathology [4, 15]. Furthermore, its application may raise concerns with evolving concepts in the treatment of inguinal hernias, as inguinal hernias have been subject to extensive research. Based on some evidence in the literature, the following factors should be taken into account to guide surgeons in their choice of treatment:

According to Matanakis’ systematic review, appendicular pathology such as a neuroendocrine tumour, appendicular pseudomyxoma, or other tumours can be diagnosed on the appendectomy specimen during the treatment of an inguinal hernia [1, 11]. This factor argues in favour of routine appendectomy or peroperative histological examination, as macroscopic appearance alone is not sufficient to rule out appendicular pathology [11]. In both of our cases, the appendix appeared inflamed macroscopically, which was confirmed by histological examination.

New hernia repair techniques, such as Desarda’s technique, have proven effective in the treatment of inguinal hernias, both during emergencies and elective surgery, regardless of the infectious context [16]. In addition, in high-income countries, biological prostheses can be used because they are more resistant to infection. They can also be placed through the laparoscopic approach [12, 17]. In our two patients, a non-absorbable prosthesis was used, precautions such as protecting the surgical site before appendectomy were taken, and povidone-iodine was instilled on the prosthesis before closure. This is a standard practice in our centre when repairing hernias with mesh. Further studies are needed to examine the benefits of these procedures.

Finally, according to the review by Matanakis, appendectomy does not appear to increase morbidity and mortality in Amyand’s hernias, even for those operated on urgently [1, 11]. In cases operated during emergencies with insertion of prosthesis, infection of the surgical site was not associated with excision of the prosthesis [1]. One of our patients presented with oedema of cord after surgery but was successfully treated with enzymatic anti-inflammatory drugs made of on trypsin, pancreatic ribonuclease and chymotrypsinogen (Ribatran^R^).

In conclusion most Amyand hernias are diagnosed during surgery for a hernia complication. There are no specific signs, hence the importance of systematically opening the hernia sac when treating a complicated hernia. During surgery, the surgeon must choose an appropriate repair technique based on the risk of infection and perform a systematic appendectomy with examination of the surgical specimen. The role of hernioplasty according to the Desarda procedure could be evaluated for this indication. The Losanoff-Basson classification must be constantly updated in line with developments in treatment procedures and available resources.

## Data Availability

The raw data supporting the conclusions of this article will be made available by the authors, without undue reservation.
